# Vector-borne diseases of small companion animals in Namibia: Literature review, knowledge gaps and opportunity for a One Health approach

**DOI:** 10.4102/jsava.v86i1.1307

**Published:** 2015-11-06

**Authors:** Bruce H. Noden, Minty Soni

**Affiliations:** 1 Department of Entomology and Plant Pathology, Oklahoma State University, United States; 2 Rhino Park Veterinary Clinic, Windhoek, Namibia

## Abstract

Namibia has a rich history in veterinary health but little is known about the vector-borne diseases that affect companion dogs and cats. The aim of this review is to summarise the existing published and available unpublished literature, put it into a wider geographical context, and explore some significant knowledge gaps. To date, only two filarial pathogens (*Dirofilaria repens* and *Acanthocheilonema dracunculoides*) and three tick-borne pathogens (*Babesia canis vogeli*, *Hepatozoon canis* and *Ehrlichia canis*) have been reported. Most studies have focused solely on dogs and cats in the urban Windhoek and surrounding areas, with almost nothing reported in rural farming areas, in either the populous northern regions or the low-income urban areas where animal owners have limited access to veterinary services. With the development of several biomedical training programmes in the country, there is now an excellent opportunity to address zoonotic vector-borne diseases through a One Health approach so as to assess the risks to small companion animals as well as diseases of public health importance.

## Introduction

Namibia consists of a large land area (823 290 km^2^) with a relatively small population (2 160 000) (CIA 2014) living in 13 regions. The northern part of the country is more humid and tropical while the central and southern regions are characterised by arid, scrubland and desert (Rohde & Hoffman 2012). Most of the human population (70%) lives in the northern one-third of the country with the rest spread into Windhoek or small urban centres scattered throughout game ranches and livestock farms in the central and southern areas (Maps, Best Country 2012). Synchronised with the climate and larger population, a higher number of Neglected Tropical Diseases (NTD) have been reported in the northern regions compared with the central and southern regions (Noden & Van der Colf 2013). In contrast, there is no published record of the distribution of dogs and cats and their specific vector-borne diseases throughout the country.

The rich history of veterinary health of Namibia has focused mostly on livestock and game farms which are important to the economy (OIE 2014; Schneider 2012). To date, very little attention has been paid to the health of small companion animals in the national research agenda (Noden & Van der Colf 2013). The only published estimate of domestic canines in Namibia is from a 2011 World Health Organization (WHO) rabies report which estimated a total number of 104 645 dogs in the country (WHO 2013). The spectrum of small companion animals in Namibia varies from well cared for pets to strays in urban areas, farm dogs and cats with minimal to no contact with veterinary services, and free-roaming populations in the lower-income areas and northern regions which have limited to no contact with their owners (Haimbodi, Mavenyengwa & Noden 2014). While the involvement of dogs and cats in zoonotic disease transmission is well documented in Africa as well as globally (Dantas-Torres 2007; Kelly *et al*. 1998; Nicholson *et al*. 2010), the lack of the most basic information in Namibia indicates that they may be directly or indirectly acting as reservoirs for infectious disease to other animals as well as human populations (Noden & Van der Colf 2013).

Gaining independence in 1990, Namibia has steadily been building its health infrastructure and lines of communication as regards infectious disease notification. However, the nascent biomedical programmes, including veterinary science, have yet to lead the discussion on vector-borne diseases of dogs and cats in the country (Noden & Van der Colf 2013). To date, most available information is found in unpublished ‘grey’ literature (theses, reports) which provides small glimpses into possible disease systems already present. These programmes could begin using this information to build an understanding of the local ecology and epidemiology of each disease. The aim of this review, then, is to summarise the existing literature (published and unpublished [theses, reports and known research]) involving vector-borne diseases of companion dogs and cats in Namibia. Discussion will include pathogens reported in small companion animals in the southern Africa region but not yet reported in Namibia. For sake of brevity, the review will not cover disease cycles, pathogenesis of diseases nor the treatment regimens which have been adequately covered elsewhere. Mention will be made of the potential risk of transmission of zoonotic pathogens between dogs and cats and human owners; however, a full review of the vector-borne pathogens that are affecting human populations in Namibia already exists (Noden & Van der Colf 2013). While an effort was made to locate relevant publications, it cannot be assumed that every study was identified. The review ends with a brief discussion of possible future directions.

## Filarial nematodes

The presence of *Dirofilaria* in Namibia has not been officially recorded in the published literature (Simon *et al*. 2012), but *Dirofilaria repens* and *Acanthocheilonema dracunculoides* have both been reported (Table 1).

**TABLE 1:** Published records of vector-borne diseases of companion dogs and cats in Namibia.

Pathogen	First recorded presence	Number animals tested or reported	Dates of reporting	Sampling area	Reference
*Dirofilaria repens*	1994	2 cats and 8 dogs	1994–2008	Central Namibia	Schwan (2009)
		5 dogs	2011	Windhoek	Noden *et al*. (2011)
*Acanthocheilonema dracunculoides*	1992	4 dogs	1992–2003	Windhoek	Schwan and Schroter (2006)
*Babesia vogeli*	2004	600 dogs	2004	Central Namibia	Stuben (2004)
		1 dog	2011	Windhoek	Noden *et al*. (2011)
*Hepatozoon canis*	2011	1 dog	2011	Windhoek	Noden *et al*. (2011)
*Ehrlichia canis*	2004	600 dogs	2004	Central Namibia	Stuben (2004)
		106 dogs	2005	Windhoek	Manyarara *et al*. (2015)

Note: Please see the full reference list of the article, Noden, B.H. & Soni, M., 2015, ‘Vector-borne diseases of small companion animals in Namibia: Literature review, knowledge gaps and opportunity for a One Health approach’, *Journal of the South African Veterinary Association* 86(1), Art. #1307, 7 pages. http://dx.doi.org/10.4102/jsava.v86i1.1307, for more information.

### Dirofilaria repens

*Dirofilaria repens* is normally associated with subcutaneous infections with limited reports of complications in southern Africa (Bredal *et al*. 1998; Schwan *et al*. 2000). Canines are considered to be reservoirs for infections with a mandatory transmission between vertebrate hosts by mosquito vectors (Simon *et al*. 2012). The frequent reports of *D. repens* as being involved in human cases together with the recent suggested involvement of cats as reservoirs (Simon *et al*. 2012) indicate a need to better understand this pathogen in its local context.

Clinical–pathological infections of *D. repens* were first recorded in central Namibia in 1994 with 80% (*n* = 8) of cases occurring in Windhoek (Schwan 2009). Between 1990 and 2007, 50% (*n* = 5) of cases were found in crossbred dogs, whereas 20% (*n* = 2) were found in domestic shorthair cats. Stuben (2004) followed by reporting the occurrence of *Dirofilaria* sp. in 3.2% of 600 canine blood smears obtained in central Namibia. Finally, in 2011, five clinical cases of *Dirofilaria* sp. out of 10 suspected dogs were identified by physical signs and blood smear at a local veterinary clinic (Noden *et al*. 2011).

*Dirofilaria* are transmitted by mosquitoes but few studies in Africa have identified with an certainty the mosquito species responsible for mode of transmission. *Aedes aegypti* has been incriminated as a vector of *D. repens* in Africa (Anyanwu *et al*. 2000; Magayuka 1973) as well as in other parts of the world (Simon *et al*. 2012). A recent survey in six locations across Windhoek determined the presence of *A. aegypti* in most low-income areas where veterinary contact is not normally available to affected animals and where the possibilities could exist for higher risk of transmission to humans (Noden *et al*. 2014a).

### Acanthocheilonema dracunculoides

*Acanthocheilonema dracunculoides* is a vector-borne apathogenic nematode involving domestic canines and some sylvatic carnivores such a spotted hyena and aardwolf (Schwan & Schroter 2006). Schwan and Schroter (2006) reported two clinical cases of *A. dracunculoides* in dogs from Windhoek. The principal intermediate hosts are the louse fly (*Hippobosca longipennis*) or the brown dog tick (*Rhipicephalus sanguineus*) which is the main tick species reported on dogs throughout central and southern Namibia (Matthee *et al*. 2010).

### Knowledge gaps

While two helminth species have been reported in Namibia, no information exists about whether *Dirofilaria immitis* or *Acanthocheilonema reconditum* (formerly *Dipetalonema reconditum*) (Nematoda: Onchocercidae) (vectored by fleas [*Ctenocephalides canis* and *Ctenocephalides felis*] and lice [*Heterodoxus spiniger* and *Trichodectes canis*]) are present in Namibian dogs and cats. Given that most reports are from arid central Namibia, there is a critical lack of epidemiological information on infections and the identity and distribution of arthropod vectors throughout the country.

## Tick-borne pathogens of companion dogs and cats

A variety of tick-borne diseases including protozoa (*Babesia* sp., *Theileria* sp., *Hepatozoon canis*) and bacteria (*Ehrlichia canis*, *Anaplasma platys*, Spotted fever group rickettsiae) have been reported in domestic canines and felines throughout southern Africa (Chitanga, Gaff & Mukaratirwa 2014). To date, only *Babesia* sp. *Hepatozoon canis* and *Ehrlichia canis* have been reported in domestic canines in Namibia (Table 1).

### Babesia

Babesiosis is an important protozoal vector-borne disease of small companion animals throughout the world (Solano-Gallego & Baneth 2011). *Babesia canis vogeli* and *Babesia canis rossi* are the only subspecies of *B. canis* to have been reported in southern Africa (Matjila *et al*. 2008b). *Babesia canis rossi* causes the most severe disease, whereas *B. c. vogeli* produces a milder, subclinical infection. *Babesia* sp. are transmitted by a variety of tick species, including *Haemaphysalis elliptica* (formerly *Haemaphysalis leachi*) (vector for *B. c. rossi*) and *R. sanguineus* (vector for *B. c. vogeli*), which are commonly reported on dogs and cats in South Africa (Bryson *et al*. 2000; Horak & Matthee 2003).

The first report of *B. canis* in Namibia occurred in the central highlands in 2004 in a study evaluating 600 dogs in low-income communities surrounding Okahandja (Stuben 2004). The seroprevalence of Babesiosis was high (69.0%) and the reported blood smear observations of sick dogs were positive for *B. c. vogeli* but parasitaemia was low (0.3%). Following this report, Noden *et al*. (2011) reported one probable case of *B. c. vogeli* in a Windhoek clinic, diagnosed by blood smear. The main vector for *B. c. vogeli* in rural, communal areas of South Africa is *R. sanguineus* which is also the principal tick species found on dogs in central Namibia (Matthee *et al*. 2010). To date, no studies have identified whether *B. c. rossi*, the more deadly form found in urban areas of South Africa (Matjila *et al*. 2008b), is also present in Namibia. Another question is whether feline Babesiosis, recently described in South Africa (Bosman, Venter & Penzhorn 2007; Bosman *et al*. 2013), also occurs in Namibia.

### Hepatozoon canis

*Hepatozoon canis* is a protozoan parasite which has a complex life-cycle that involves ticks (Baneth, Samish & Shkap 2007). In southern Africa, *H. canis* has been reported in wild felines and canids (Matjila *et al*. 2008a; Williams *et al*. 2014). The main vector species in southern Africa is considered to be *R. sanguineus*, but, to date, *H. canis* has yet to be found in *R. sanguineus* collected in sub-Saharan Africa. In Namibia, only one case of *H. canis* has been recorded in a dog diagnosed in a veterinary clinic in urban Windhoek that was based only on signs and symptoms and a blood smear (Noden *et al*. 2011).

### Ehrlichia canis

*Ehrlichia canis* is a globally important pathogen affecting canines (Harrus & Waner 2011) that has been reported in Zimbabwe and South Africa by seroprevalence studies. The main tick vector is assumed to be *R. sanguineus* (Matthewman *et al*. 1997a).

The first published record of *E. canis* in Namibia was by Stuben (2004) who noted clinical symptoms in 23.5% of 600 dogs studied in a low-income area around Okahandja in central Namibia. 14.2% had evident morulae in their blood smear analysis. The incidence of ehrlichiosis was significantly higher in dogs with poor nutritional status, poor grooming and heavy tick infestation.

This work was followed by Manyarara *et al*. (2015) who demonstrated a canine seroprevalence against *E. canis* using an ELISA-based assay. Dogs presenting at a clinic with signs of *E. canis* infection had a significantly higher seroprevelence (86.6%) compared with other dogs (41.6%). The location of habitation was significant, with a high percentage of *E. canis*-exposed dogs living in the northern or northwestern part of Windhoek. As the first study to establish *E. canis* serologically as a significant pathogen to dogs in central Namibia, it was notable that the highest proportion of positive dogs came from the low-income areas. It is assumed that the principal tick vector in Namibia is *R. sanguineus* as this is the primary tick recovered from dogs in veterinary clinics in central and southern Namibia (Matthee *et al*. 2010), but this has yet to be confirmed.

### Knowledge gap

To date, there is limited to no information in Namibia concerning the following tick-borne pathogens which have been reported elsewhere in southern Africa.

#### *Theileria* sp. and *Anaplasma platys*

Whereas both have been reported in South African dogs (Inokuma *et al*. 2005; Matjila *et al*. 2008c), these pathogens have yet to be reported in Namibia.

#### Spotted fever group rickettsiae

Tick-transmitted spotted fever group (SFG) rickettsiae in southern Africa mainly involves *Rickettsia conorii* and *Rickettsia africae* (Pretorius, Jensenius & Birtles 2004). The role of dogs and cats as reservoirs for these pathogenic bacteria was hypothesised in southern Africa studies in the 1990s (Kelly & Mason 1991; Matthewman *et al*. 1997a). Recently, domestic canines been implicated as reservoirs of SFG rickettsiae in many areas of the world, except in Africa (Izzard *et al*. 2010; Ortuno *et al*. 2009). The presence of SFG rickettsiae species has not yet been reported in Namibian dogs and cats; however, serological evidence has been reported in human populations (Noden & Van der Colf 2013; Noden *et al*. 2014b). While the main tick reservoir or vector species in Africa for *R. conorii* and *R. africae* is probably *R. sanguineus* (Walker *et al*. 2007), the potential involvement of seven other known Namibian tick vector species remains unknown (Noden & Van der Colf 2013).

#### *Coxiella burnetii* (Q fever)

*Coxiella burnetii*, the causative agent for Q fever, is underreported in southern Africa. Normally associated with cattle, sheep and goats (Kelly *et al*. 1993; Magwedere *et al*. 2012), seroprevalence has been reported for cats and dogs from Zimbabwe and South Africa (Kelly *et al*. 1993; Matthewman *et al*. 1997b). Normally, exposure to *C. burnetii* occurs by aerosol or milk products, but ticks also act as reservoirs (Kelly *et al*. 1993). Five species of Namibian tick could be reservoirs or carriers of *Coxiella* (Noden & Van der Colf 2013), but the involvement of companion dogs and cats remains unknown (Magwedere *et al*. 2012).

## Flea-borne pathogens of companion animals

### Flea-borne rickettsiae

*Rickettsia typhi* (typhus group) and *Rickettsia felis* (spotted fever group) are rickettsiae of public health importance transmitted by fleas (Azad *et al*. 1997). Normally not associated with companion animals, recently dogs and cats were implicated as reservoirs for infection in Europe, thus posing a risk for zoonotic transmission to human populations (Nogueras *et al*. 2013a, b). Serological evidence for both *Rickettsia* species has been recorded in cats and dogs from Zimbabwe and South Africa (Matthewman *et al*. 1997a). The main vector species of these bacteria in Africa is the rat flea, *Xenopsylla cheopis*, and the cat flea, *C. felis* (Azad *et al*. 1997; Laudisoit *et al*. 2014).

### Cat scratch fever

*Bartonella henselae* is another flea-borne bacteria of public health importance with zoonotic transmission potential reported in dogs, cats and humans in South Africa (Maggi *et al*. 2013; Pretorius *et al*. 1999), Zimbabwe (Kelly *et al*. 1998) and Namibia (Noden *et al*. 2014b). Vectored by the cat flea, *C. felis*, or the rat flea, *X. cheopis* (Laudisoit *et al*. 2014), infected cats and their fleas have been identified as a significant risk factor for patients infected with HIV in South Africa (Frean, Arndt & Spencer 2002).

### Gap analysis

Similarly to tick-borne diseases, there is a dearth of information in Namibia and the southern Africa region concerning the ecology of fleas on small companion animals, the pathogens they carry and their possible zoonotic impact in veterinary and public health. The relatively high seropositivity rates in healthy human blood donors (Noden *et al*. 2014b) suggest that infected fleas exist in close proximity to pet owners, but few veterinarians in Namibia even mention fleas on dogs and cats. Further study is needed to identify areas of Namibia most prone to domestic flea infestations and the possibility of flea-borne rickettsiae among canine and feline populations and local flea populations.

## Important pathogen of public health importance with possible canine involvement

Leishmania is an NTD which annually affects millions of domestic dogs and cats and humans around the world (Pennisi 2015). It has been reported in the arid areas of southern Africa (South Africa [Rutherfoord & Uys 1978], Namibia [Grove 1989], and Botswana [Schwartz *et al*. 2012]). Both the cutaneous and visceral forms are transmitted by sandflies and the natural reservoirs are jackals, dogs and hyrax, with humans serving as accidental hosts. In recent years, the influence of dogs or cats, particularly rural or shelter canines, as reservoirs of *L. infantum* and other *Leishmania* species has been increasingly recognised in South America and Europe (Dantas-Torres 2007; Pennisi 2015; Rougeron *et al*. 2011), with a few reported cases in north and west Africa (Berdjane-Brouk *et al*. 2012; Guessous-Idrissi *et al*. 1997). Nothing, besides historical reports and one recent tourist case study, is known about the ecology and distribution of this important pathogen in southern Africa.

Autochthonous cutaneous Leishmania (CL), aligned with the *Leishmania tropica* group, has been reported in Namibia since the 1970s (Grove 1989). Most of the CL cases between 1970 and 1989 were geographically located in the southern and central regions and linked to a history of picnicking, camping or living near hyrax (*Procavia capensis*) colonies. Although no human clinical data has been published since 1989, clinical cases involving both canines (OIE 2014) and humans (Noden, unpublished data 2012) continue to be reported in Namibia.

Normally, there is not much focus on autochthonous CL cases involving rural dogs or humans as the risk is considered low. However, as events in southern Namibia in the early 1970s demonstrated, if autochthonous CL is not investigated, it can suddenly become an epidemic should ecological and environmental conditions change (Noden & Van der Colf 2013). In the mid-1970s, a small CL outbreak occurred among humans in southern Namibia during a period when hyrax populations (the reservoir hosts) were exploding because of good rains and the lack of a predator. The Verreaux's (Black) eagle populations had significantly diminished because sheep farmers believed they were targeting their lambs, when in actual fact the eagles almost exclusively prefer the hyrax. This concurrent event coincided with humans camping and fishing near hyrax burrows which contained infected sandflies looking for blood meals. The fact that *Leishmania* continues to be reported vaguely in the OIE biannual reports in Namibia and occasional human encounters involving stories of a non-healing insect bite suggest that CL is probably still present and is being locally transmitted by sandflies. However, nothing is known about its epidemiology or the risks of acquiring infection. The use of domestic dogs on area farms as sentinels throughout the central and southern regions of Namibia would provide an excellent means of determining potential ‘hot spots’ where a zoonotic infection could spill over into human populations. While Leishmania is prone to outbreaks that could affect the tourism industry, often infections in dogs are often not curable, which results in significant morbidity and a drawn-out period until death.

## Points to consider

### Go beyond the urban areas of central Namibia

Most of the Namibia-based studies summarised in this review focused solely on dogs and cats in urban areas with relatively affluent owners working with established veterinary practices from Windhoek and the immediate surrounding central regions (Table 1). This is convenience sampling at best. As in the case of Leishmania, little to nothing is known about what dogs and cats are exposed to in rural farming areas, in the populous northern regions or in the low-income urban areas where animal owners have limited access to veterinary services or no money to pay for them. There is a need for the developing veterinary health programme to focus on the whole country when evaluating the epidemiology of each pathogen in order to gain a holistic understanding of its unique ecology and distribution within Namibia.

### One Health assessment of vector-borne disease epidemiology in Namibia

#### Use of sentinel animals to focus on diseases of public health importance

The One Health paradigm is a way to consider zoonotic diseases in a holistic way, focusing on the environment, animals, and humans, by addressing specific aspects of disease epidemiology at a reduced cost (Okello *et al*. 2014; Yancey *et al*. 2014). One globally accepted idea is the use of dogs and cats as sentinel animals in public health surveillance efforts (Schmidt 2009; Schurer *et al*. 2012). By strategically focusing on particular areas in the country to reduce costs, blood samples could be obtained from companion dogs and cats instead of people. These could then be tested for exposure to various vector-borne pathogens of importance to both veterinary and public health (Yancey *et al*. 2014) (Table 2). This method has been used in other countries to monitor exposure rates in local areas for many vector-borne diseases (De Paiva Diniz *et al*. 2007; Izzard *et al*. 2010; Ortuno *et al*. 2009; Resnick *et al*. 2008; Schurer *et al*. 2012; Smith *et al*. 2012).

**TABLE 2:** Vector-borne diseases of public and veterinary health importance reported in Namibia for which surveillance could be enhanced using sentinel studies in companion dogs and cats.

Disease	Pathogen name	Vector	Probable animals to test	Supporting reference
Dirofilaria	*Dirofilaria repens*	Mosquito	Dogs	Simon *et al*. (2012)
Leishmania	*Leishmania major* group	Sandfly	Farm dogs	Pennisi (2015)
Crimean Congo Hemorrhagic fever	CCHF virus	Tick	Dogs and cats	Shepherd *et al*. (1987)
Anaplasmosis	*Anaplasma platys*	Tick	Dogs	Sanogo *et al*. (2003)
Rickettsiosis	*Rickettsia conorii/ Rickettsia africae*	Tick	Dogs and cats	Ortuno *et al*. (2009)
	*Rickettsia typhi/ Rickettsia felis*	Flea	Dogs and cats	Nogueras *et al*. (2013a, b)
Bartonellosis	*Bartonella henselae*	Flea	Dogs and cats	Pretorius *et al*. (1999)
Borreliosis	*Borrelia* sp.	Tick	Dogs and cats	Smith *et al*. (2012)

Note: Please see the full reference list of the article, Noden, B.H. & Soni, M., 2015, ‘Vector-borne diseases of small companion animals in Namibia: Literature review, knowledge gaps and opportunity for a One Health approach’, *Journal of the South African Veterinary Association* 86(1), Art. #1307, 7 pages. http://dx.doi.org/10.4102/jsava.v86i1.1307, for more information.

For example, a One Health approach focusing on farm dogs and cats could be used to provide critical information regarding the Crimean Congo Hemorrhagic Fever virus (CCHFV), an extremely dangerous tick-borne virus that occasionally occurs in Namibia. Even through infrequent cases indicate the virus is autochthonous in the country (Noden & Van der Colf 2013), it is a BSL-4 level virus and an outbreak could have a critical impact on the agricultural and tourism sectors. To date, almost nothing is known about the ecology and epidemiology of this lethal zoonotic pathogen in Namibia (Noden & Van der Colf 2013). The use of serological tests from companion dogs or cats on farms could provide useful epidemiological data as to potential ‘hot-spots’ in the central and southern regions to which more effort could be directed to study the ecology, vectors and risk potential. Such an approach was taken in the mid-1970s to describe the epidemiology of CCHFV in South Africa using a variety of serum samples from various mammals, including domestic dogs (Shepherd *et al*. 1987). An exposure prevalence of 6% was reported in 1978 canine samples tested from around South Africa.

## Conclusion

In conclusion, to date, only five vector-borne pathogens affecting domestic dogs and cats have been reported in Namibia. However, many others are known to occur within the southern Africa region, some of which are zoonotic and can spread to human populations. As highlighted, there is much work to be done by nascent training programmes throughout the region. In addition to monitoring the impact of other pathogens on small companion animals in rural and urban areas, there is a need to verify the arthropod vectors of these diseases. The useful development of a One Health paradigm between the veterinary and public health services could assist in effectively tracking the epidemiology of these important pathogens in small companion animals as well as in the public health domain.
